# High-dose X-ray radiation induced MgO degradation and breakdown in spin transfer torque magnetic tunnel junctions

**DOI:** 10.1038/s41598-022-19342-x

**Published:** 2022-11-03

**Authors:** Qi He, Hui Shi, Yinquan Wang, Lichao Cao, Xiang Gu, Jianwei Wu, Genshen Hong, Minghua Li

**Affiliations:** 1 The 58th Research Institute, China Electronics Technology Group Corporation, Wuxi, 214072 China; 2 Department of Material Physics and Chemistry, School of Material Science and Engineering, University of Science and Technology Beijing, Beijing, 100083 China; 3 Beijing Laboratory of Metallic Materials and Processing for Modern Transportation, University of Science and Technology Beijing, Beijing, 100083 China

**Keywords:** Astronomy and planetary science, Materials science, Nanoscience and technology

## Abstract

Magnetic tunnel junction (MTJ) with magnesium oxide (MgO) tunnel barrier is the core element of spin transfer torque-based magnetic random access memory. For the application in the space environment, the total ionizing dose radiation effects on MTJs need to be evaluated. In this work, the MTJs were exposed to X-ray radiation with different doses of up to 10 Mrad(Si). Measurements of current induced magnetization switching (CIMS) behavior of these MTJs were performed before and after radiation. The results show negligible changes in the tunneling magnetoresistance and current switching properties after 8 Mrad(Si) X-ray radiation. However, with a total dose of 9 Mrad(Si), a significant reduction in junction resistance of a fairly large number of MTJs was observed, which showed characteristics of MTJ breakdown. Moreover, in this study, all experimental MTJs became functionally disabled due to MgO breakdown under 10 Mrad(Si) X-ray radiation. The CoFeB/MgO/CoFeB interface microstructure was observed using X-ray photoelectron spectroscopy and high-resolution transmission electron microscopy (HRTEM). Interfacial structural results indicate that the MgO degradation and breakdown behavior caused by X-ray ionizing radiation can give rise to radiation-induced oxygen vacancies across the tunnel barrier oxide layer.

## Introduction

Magnetic random access memory, which is based on CMOS peripheral circuit and memory cell arrays, possesses strong robustness against radiation effects, lower power consumption, fast speed, and high density and has a great prospect in aeronautics and astronautics application^1–3^. In general, space radiation effects on electronic systems mainly include total ionizing dose effects (TID) and single event effects (SEE). Previous studies have been carried out on the TID effects of both the first generation MRAM^4,5^ (Toggle-MRAM) and the second-generation MRAM^6,7^ (STT-MRAM) chips. These studies indicate that the soft or hard errors of MRAM are mainly attributable to the CMOS sense circuit because it is sensitive to TID. The typically adopted MRAM memory cell consists of one access transistor and one magnetic tunnel junction (MTJ). For the access transistor, the pioneering work^8^ shows that TID radiation could induce resistance shift of the access transistor, which is also responsible for the upset errors in MRAM. With regard to MTJ, the structure of MTJ usually includes two ferromagnetic layers separated by an oxide layer (i.e. tunnel barrier). The junction resistance is dependent on the relative orientation of the magnetization of the two ferromagnetic layers. This physical phenomenon is called the tunneling magnetoresistance (TMR) effect. For the first Toggle-MRAM, the oxide layer is amorphous AlOx and for STT-MRAM is crystalline MgO. It is pointed out that MTJs with a single-crystalline MgO(100) barrier could achieve a giant TMR up to 1000% in the theoretical calculation^9^ and 604% in experimental observation^10^. With the rapid scaling of process nodes and the popular application of STT-MRAM instead of Toggle-MRAM, there has been a great interest in TID radiation effects on MgO-based magnetic tunnel junctions^11^. Ren et al.^12^and Hughes et al.^13^ respectively studied the cobalt-60 gamma-ray radiation tolerance of micrometer-scale and nanometer-scale MTJs with in-plane magnetic anisotropy (i-MTJs). Both studies concluded that ionizing radiation has a negligible impact either on TMR or on field-induced switching properties of i-MTJs. Recently, Montoya et al.^14^ characterized the current-induced switching property of nanoscale MTJs with perpendicular magnetic anisotropy (p-MTJs) before and after gamma radiation, which also demonstrated that all key properties of p-MTJs are robust against ionizing radiation of cobalt-60 gamma-ray. It has been pointed out that gamma-ray radiation is involved more in electron–hole pair creation rather than in trap generation. However, cosmic rays include γ-ray and X-ray, both causing degradation or even failure of electronic devices. Since the X-ray radiation effect on MTJs is unknown, in the present work, we have chosen the nanoscale MTJs to experiment.

In this work, we report the X-ray radiation experiments of the MgO-based MTJs up to 10 Mrad(Si) total doses. The results show that negligible changes were observed in the tunneling magnetoresistance (TMR) and current switching properties after 8 Mrad(Si) X-ray radiation. However, after 9 Mrad(Si) radiation, even a complete CIMS curve of many MTJ samples cannot be obtained. Moreover, a significant reduction in junction resistance occurred due to MgO instability during the curve sweep. With the radiation dose reaching 10 Mrad(Si), the study finds that all experimental MTJs have experienced MgO breakdown during radiation. We demonstrate that the MgO degradation and breakdown caused by X-ray ionizing radiation is owing to radiation-induced oxygen vacancies in the oxide. Our results show an important step towards the understanding of TID radiation tolerance of nanoscale MTJs for space application in novel STT-MRAM devices.

## Results and discussion

**Figure 1:**
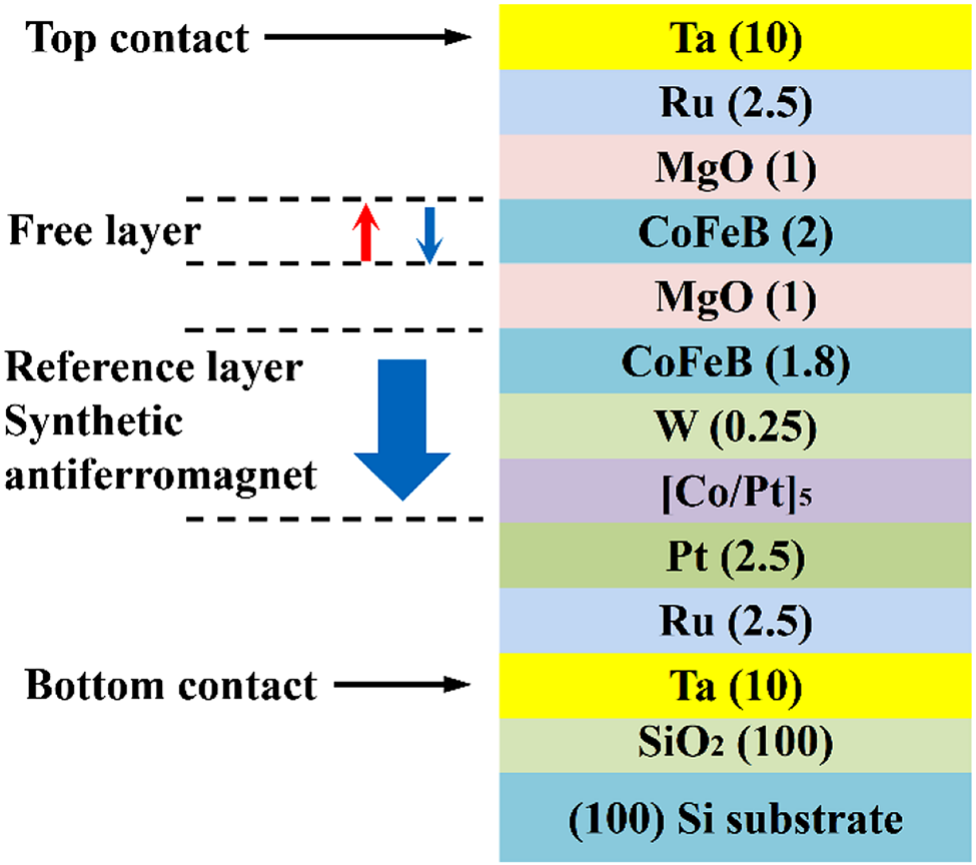
Structural schematic diagram of the spin transfer torque film stacks. Numerical numbers in parentheses represent the nominal thickness of each layer in nanometers except for (100) Si substrate crystal orientation.

The STT film stack forms the basis for STT-MTJ fabrication used in this study is shown in Fig. 1. The base is composed of a (100) silicon substrate and a thermally grown oxide, typically 100 nm. The MTJ layer sequence is Ta (10)/Ru (2.5)/Pt (2.5)/[Co/Pt]_5_/W (0.25)/CoFeB (1.8)/MgO (1) /CoFeB (2)/MgO (1)/Ru (2.5)/Ta (10) (unit: nm). After the deposition of the multifilm stack, the MTJ structures were fabricated using photolithography and ion milling. The final shape of MTJs is elliptical and the size is 60 × 150 nm^2^ (minor × major axes). Details of the MTJ fabrication procedure are described in the “Methods” section.

**Figure 2:**
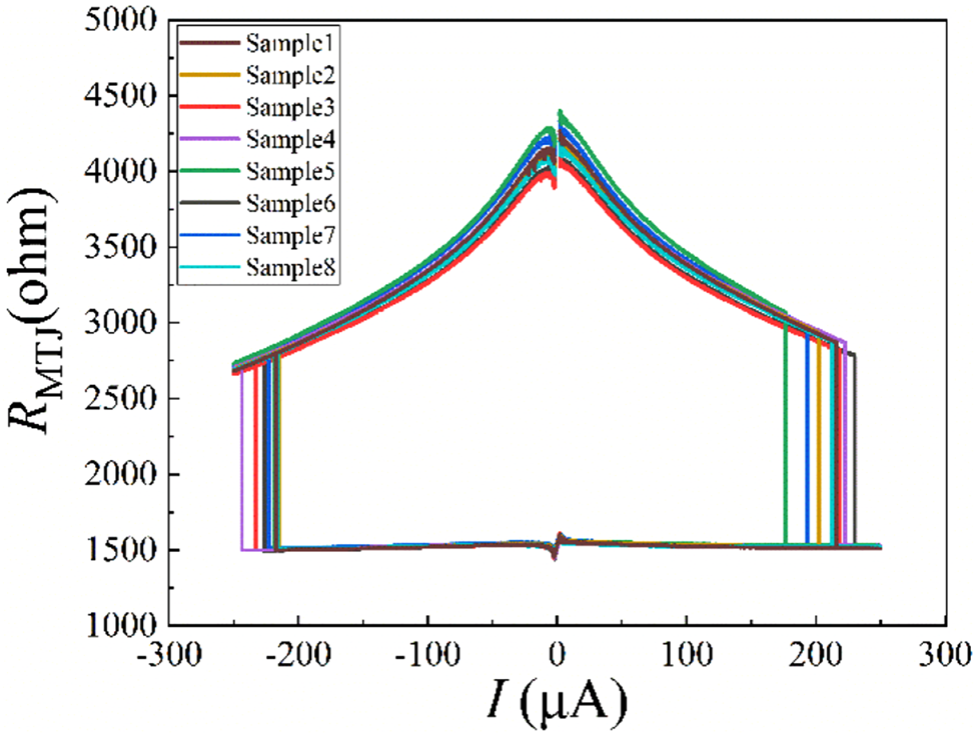
Current induced magnetization switching characteristics of MTJ devices before radiation. Sample 1 ~ sample8 are randomly selected eight devices.

As is shown in Fig. 2, the current-induced magnetization switching characteristics of eight MTJ devices (randomly selected and named Sample1 to Sample8) in the experimental group were performed by Semiconductor Device Analyzer B1500 before exposure to X-ray radiation. The x-axis represents the current into the pad connected to the free layer of the MTJ device and the CIMS loops are clockwise. When the current reached around 220 uA, the loop switched from high resistance to low resistance (AP to P); on the contrary, the loop switched from low resistance to high resistance (P to AP) when it reached about − 220 uA. It can be obtained by a simple calculation that the critical current density is approximately 3.1 × 10^6^ A/cm^2^, which is consistent with the previous reports^15^. However, there are few deviations among the testing devices in terms of TMR, bias-voltage dependence of TMR, and critical switching current (*I*_c_). This is because of the process variation during MTJ device fabrication. The TMR values and critical switching currents of sample1 ~ 8 are listed in Table 1.

**Table 1:** TMR ratio and critical switching current of MTJ devices (sample1 ~ sample8) before radiation.

NO	TMR ratio	Ic (AP to P)	Ic (P to AP)
Sample1	170.8%	216 uA	−217 uA
Sample2	167.9%	203 uA	−214 uA
Sample3	159.5%	218 uA	−233 uA
Sample4	169.9%	223 uA	−243 uA
Sample5	179.1%	175 uA	−217 uA
Sample6	162.7%	229 uA	−226 uA
Sample7	174.5%	193 uA	−222 uA
Sample8	167.3%	212 uA	−216 uA

**Figure 3:**
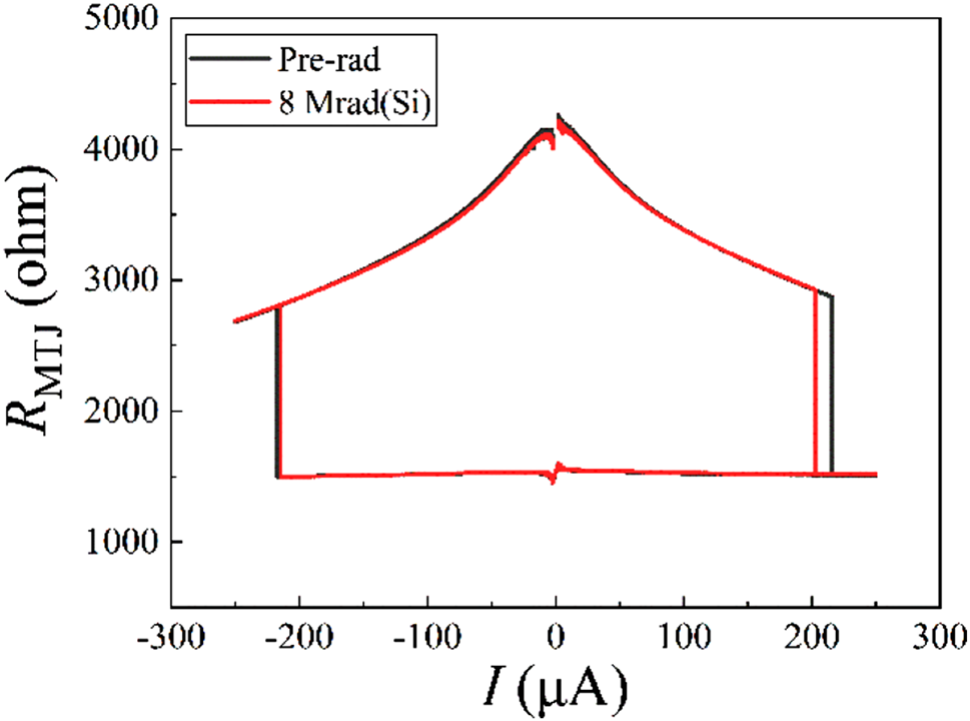
Current induced magnetization switching characteristics of a typical MTJ device before radiation and after 8 Mrad(Si) X-ray radiation. Sample 1 ~ sample8 are randomly selected eight devices.

As is shown in Fig. 3, the current-induced magnetization switching characteristics of a typical MTJ device were tested before (Pre-rad) and after X-ray radiation exposure of 8 Mrad(Si). There is only a little difference between critical switching currents from AP to P before and after 8 Mrad(Si) radiation. The critical switching currents from P to AP and TMR ratios are almost the same. The results indicate that 8 Mrad(Si) X-ray radiation has little influence on the switching properties of MTJs.

**Figure 4:**
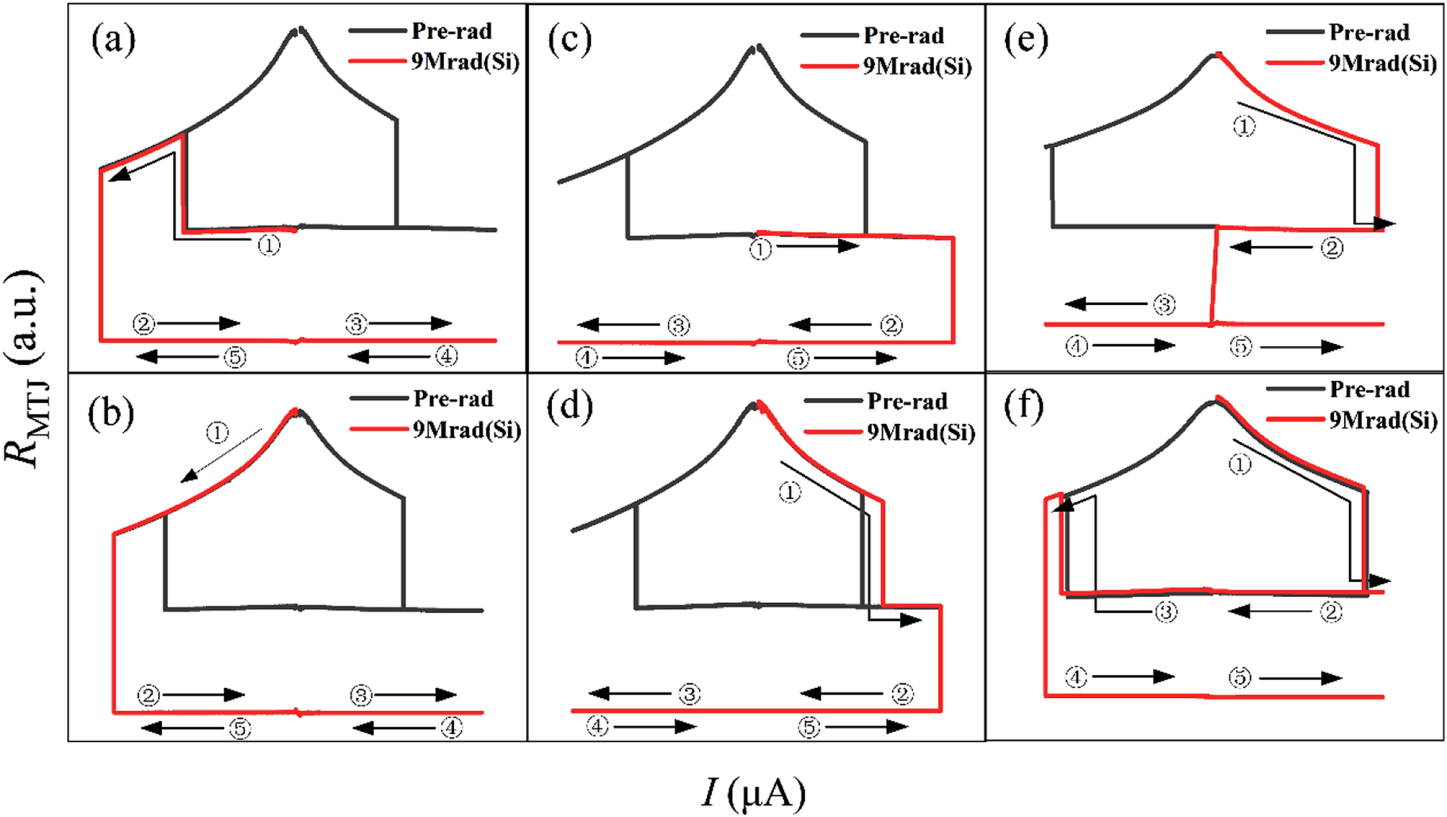
Current induced switching characteristics of MTJ devices before and after 9 Mrad(Si) total ionizing dose radiation with different initial sweep directions and different initial resistance. (**a**) negative sweep from initial low resistance state, (**b**) negative sweep from initial high resistance state, (**c**) positive sweep from initial low resistance state, (**d**) positive sweep from initial high resistance state, (**e**) positive sweep from initial high resistance state, (**f**) CIMS curves remained complete if sweep region shrank.

The CIMS characteristics of all MTJ devices were tested before and after X-ray radiation exposure of 9 Mrad(Si). Some MTJs still show similar switching properties as those of Pre-rad, which is strong evidence that magnetic tunnel junctions are robust to total ionizing dose radiation. However, more MTJs exhibit breakdown characteristics during the curve sweep as shown in Fig. 4. As mentioned before, the CIMS curve of MTJ devices was characterized by Semiconductor Device Analyzer B1500. The complete switching procedure consists of five parts. For the MTJs before radiation, the initial sweep direction is positive, and the first part of the procedure sweeps current from 0 to the maximum positive current (donated as *I*_max_). Then the next part is from *I*_max_ to 0. The third part of the procedure sweeps the current from 0 to the maximum negative current (donated as *I*_min_). The two remaining parts are current sweeps from *I*_min_ to 0 and 0 to *I*_max_. The sweep current interval is set as 100 nA for positive sweep direction and − 100 nA for negative sweep direction. To reveal the breakdown characteristic of MTJs after 9 Mrad(Si) radiation, we also choose the initial negative sweep direction and the switching procedure is comprised of five current sweeps as follows: 0 to *I*_min_, *I*_min_ to 0, 0 to *I*_max_, *I*_max_ to 0, and 0 to *I*_min_. Figure 4a and b are the cases where the initial sweep direction of current is negative. Regardless of the initial MTJ resistance, the CIMS curve only runs the first part of the testing procedure after 9 Mrad(Si) radiation. Running the remaining four parts of the procedure can only get about one hundred ohms for MTJ resistance, which is the breakdown characteristic of MTJ breakdown. Figure 4c to f show the cases when the initial current direction is positive. The difference lies in that Fig. 4c and d run a quarter CIMS curve after radiation while two quarters for Fig. 4e and three quarters for Fig. 4f. All above incomplete CIMS curves indicate that MgO becomes deteriorated and unstable after 9 Mrad(Si) radiation.

**Figure 5:**
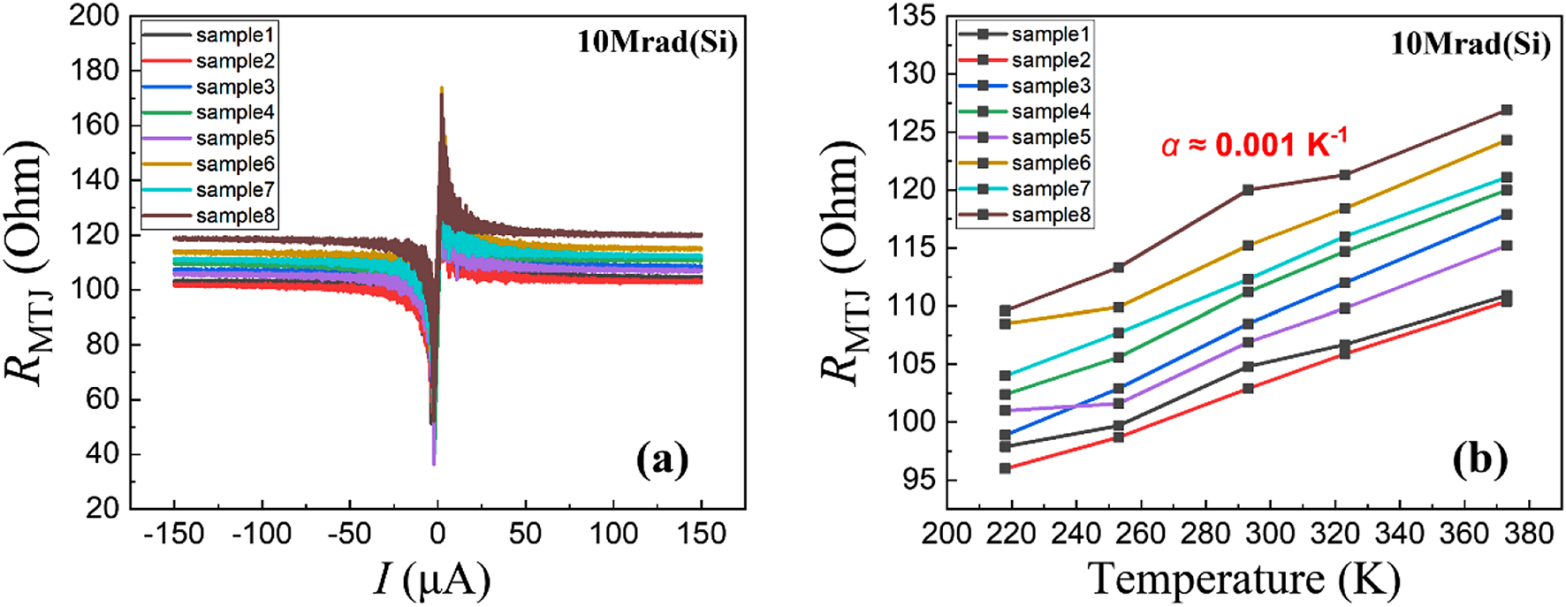
(**a**) Current induced switching characteristics and (**b**) temperature dependence of resistance of MTJs (sample1 ~ sample8) after radiation with 10 Mrad(Si) total ionizing dose.

After 10 Mrad(Si) X-ray radiation exposure, all experimental MTJs show breakdown characteristics. Figure 5a shows the R-V characteristics of MTJ devices from sample1 to sample8. There are peaks of the resistance around zero, which is usually caused by an offset such that there is some offset current if the voltage is zero. The MTJ resistances all fall to around 100 ohms, which is close to the resistance value after MTJ direct breakdown without radiation (not shown). We speculate that after 10 Mrad(Si) radiation, the breakdown phenomenon of the MgO tunnel barrier has occurred.

Then the temperature dependence of MTJ resistance is studied and the thermal coefficients of resistivity (α) are calculated for sample 1 to sample 8 (shown in Fig. 5b). The positive thermal coefficients of resistivity indicate the metal-like conductive property of MgO after 10 Mrad(Si) radiation^16^. According to the literature^17^, the thermal coefficients of resistivity of Mg, Co, Fe, and Ta are 0.0165 K^−1^, 0.00604 K^−1^, 0.00651 K^−1^, and 0.008 K^−1^ respectively, while the thermal coefficients of resistivity are very small and about 0.001 K^−1^ after 10 Mrad(Si) radiation. For an ideal metal with a pure crystal structure, its resistivity comes from the scattering of electrons in the lattice structure and is strongly dependent on temperature. The resistivity formed by the scattering of electrons over the defect is temperature independent. In the absence of any defects, the thermal coefficient of resistivity has a theoretical maximum. Thus the ultra-small α value implies that the conducting paths in the MgO consist largely of radiation-induced defects instead of metal atoms diffusing from the adjacent layers.

**Figure 6:**
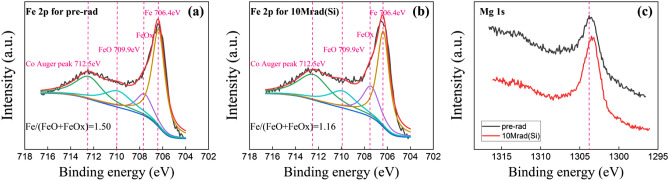
(**a**) Fe 2p XPS spectrum before X-ray radiation, (**b**) Fe 2p XPS spectrum after radiation with 10 Mrad(Si) total ionizing dose, and (**c**) Mg 1 s XPS spectra without radiation and with 10 Mrad(Si) radiation. All above XPS spectra are detected from the MgO/CoFeB interface in the Ta/MgO/CoFeB/Ta film.

XPS is one of the most widely used tools for investigating the interfacial state of materials. We carefully performed an etching process on two kind of multilayer thin-film samples with the following layer sequences: (1) Ta (10)/MgO (1)/CoFeB (2)/Ta (2) (unit: nm)and (2) Ta (10)/CoFeB (1.8)/MgO (1)/Ta (2) (unit: nm). The XPS detectable sample depth could be calculated using *d* = 3*λ*sin*ɵ*, in which *λ* is the inelastic mean-free paths (IMFPs) for the photoelectrons and *ɵ* is a take-off angle for photoelectrons with respect to the sample surface (*ɵ* = 90° in this study). The inelastic mean free paths of Mg 1 s electron and Fe 2P electron are 0.81 and 1.36 nm respectively. The inelastic mean free paths of Mg 1 s electron and Co 2P electron in their corresponding oxides are generally 0.1 ~ 0.2 nm higher than that in their metallic states. Thus, the detection depths of elemental Mg 1 s electron and Fe 2p electron are 2.43 and 4.08 nm, respectively. Therefore, to detect the electronic structure information at the MgO/CoFeB interface, it is necessary to etch part of the top metal layer with an Ar^+^-ion gun. Figure 6a and b shows high-resolution Fe 2p XPS spectra and the computer-fitted curves of the Ta/CoFeB/MgO/Ta multifilm after etching for 20 s (Ta was etched off by about 1.5 nm), which corresponds to the CoFeB/MgO interface. According to XPS Handbook^18^, the peaks of Fe 2*p*_3/2_, FeO_x_ (x < 1) 2*p*_3/2_, FeO 2*p*_3/2_, and Co Auger peak are located at 706.4, 708.8, 709.9, and 712.5 eV, respectively. We believe that 712.5 eV is the Auger peak of Co rather than the Fe_2_O_3_ 2*p*_3/2_ peak because we don’t find the Fe_2_O_3_ 2*p*_1/2_ peak near 724 eV, which indicates that the content of Fe^3+^ is very low. In addition, with the etching time increasing from 0 s, the peak intensity continues to increase, indicating that the interior of CoFeB film has been detected. At this time, the content of elemental Co and Fe is the main, so the Auger peak (712.5 eV) intensity of Co continues to increase. The presence of FeO 2*p*_3/2_ peak indicates that Fe atoms at MgO/CoFeB interface are partially oxidized to FeO due to the diffusion of O atoms during MgO growth. The Fe–O content in CoFeB can be estimated by the peak area ratio of S_Fe_/(S_FeO_ + S_FeOx_). As shown in Fig. 6a and b, the peak area ratios are 1.50 before radiation and 1.16 after radiation. As seen in Fig. 6c, the Mg 1 s peak values before and after radiation are 1304.9 and 1303.4 eV, respectively. So after 10 Mrad(Si) dose X-ray radiation, the peak of Mg 1 s decreases by 0.5 eV, indicating that O atoms diffused from the interior of MgO to the CoFeB /MgO interface and oxygen vacancies are generated at the top of MgO, which is consistent with previous reports^19–21^.

**Figure 7:**
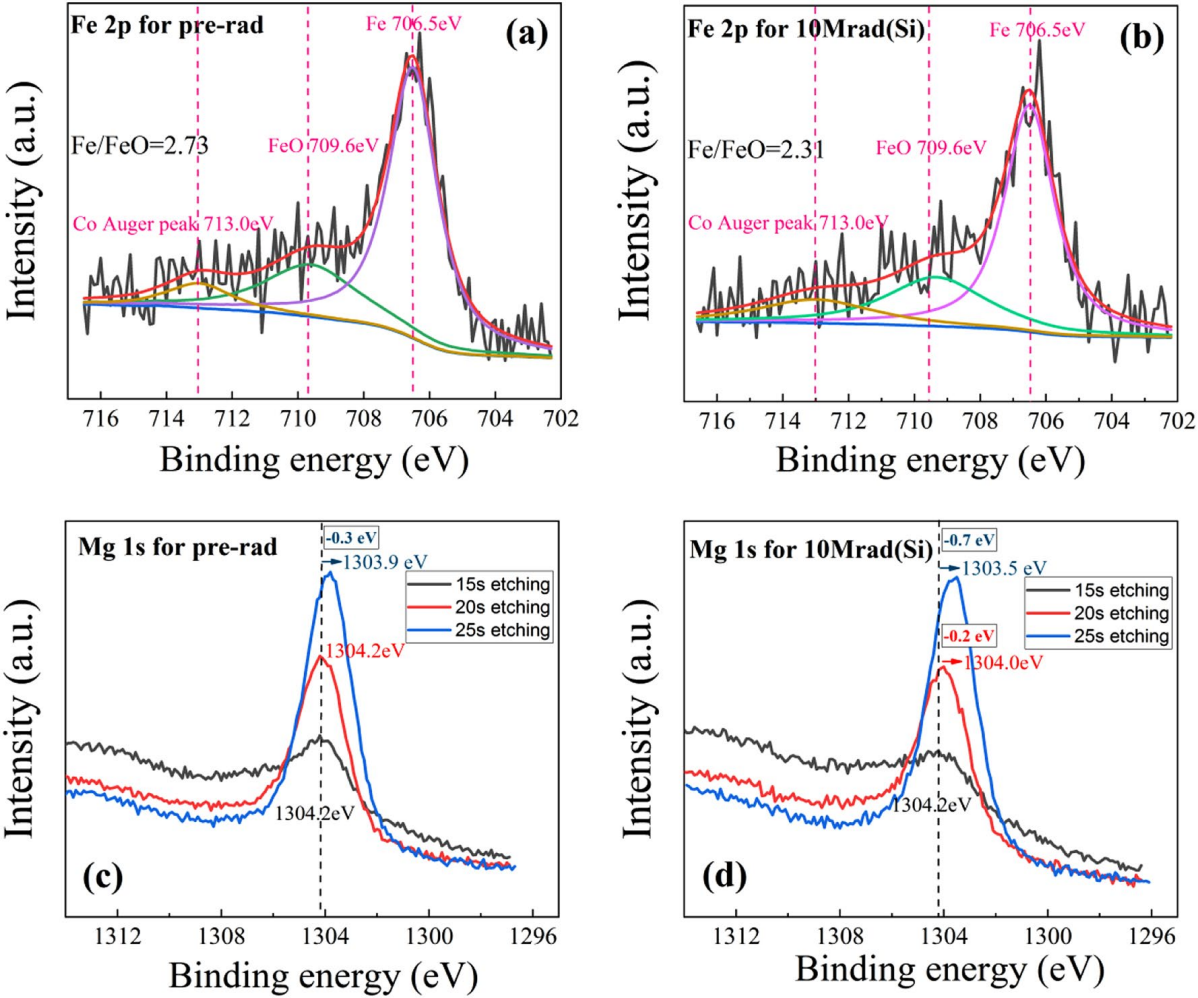
(**a**) Fe 2p XPS spectrum before X-ray radiation, (**b**) Fe 2p XPS spectrum after radiation with 10 Mrad(Si) total ionizing dose, (**c**) Mg 1 s XPS spectra before X-ray radiation at different etching times, and (**d**) Mg 1 s XPS spectra after 10 Mrad(Si) radiation at different etching time. Fe XPS spectra are detected from the CoFeB/ MgO interface in the Ta/CoFeB/MgO/Ta film. Mg XPS spectra are detected from the top of the MgO layer to the CoFeB/ MgO interface in the Ta/CoFeB/MgO/Ta film.

Figure 7 shows the Fe 2p spectra at the CoFeB/MgO interface and Mg 1 s spectra at different etching times in the Ta/CoFeB/MgO/Ta film. After 15 s etching (Ta was etched off by about 0.5 nm), we detected the peak signal of Fe 2p, indicating that the detection depth is just near the CoFeB /MgO interface. After 25 s etching, the Mg 1 s signal begins to weaken slightly, indicating that the etching depth was just at the upper interface of MgO. These peak positions are close to but not the same as the previous samples. This is because, in the Ta/CoFeB/MgO/Ta film, the Fe element was just detected at the interface. FeO content and Co content were small, so the peak shape is relatively flat. So it’s normal to have some differences among the peak positions of Fe 2*p*_3/2_, FeO 2*p*_3/2_, and Co Auger peak between two XPS samples. However, the peak position is fixed when the same sample is fitted before and after radiation. It can be seen that the Fe/FeO area ratio is 2.73 before radiation and 2.31 after radiation. This indicates that the O content in the CoFeB /MgO interface increases after radiation.

At the 15 s etching time, the Mg 1 s spectrum is mainly detected from the top of MgO. At this time, the intensity of Mg 1 s is not too strong, and the peak position is at 1304.2 eV. At the 20 s etching time, the interfacial information of Mg 1 s is detected from the top and center of MgO. At this time, the intensity of Mg 1 s increases and the peak is also 1304.2 eV. At the 25 s etching time, the Mg 1 s spectrum detects all the Mg 1 s information of the MgO layer. At this time, the intensity of Mg 1 s is the highest, with a peak of 1303.9 eV, 0.3 eV lower than the top of MgO. Since the Mg 1 s signal at this time is jointly contributed by the Mg 1 s signal at the top, middle, and bottom of the MgO layer, and the peak of Mg 1 s at the top and middle of MgO is both 1304.2 eV, it implies that the Mg 1 s peak at the bottom of the MgO layer may be lower than 1303.9 eV. Therefore, the distribution of O before radiation is that there are fewer O atoms at the bottom of the MgO and CoFeB /MgO interface, while there are more O atoms at the top and middle of MgO. According to the XPS manual, the binding energy of Mg 1 s in stoichiometric MgO is located at 1303.80 eV, so the MgO content ratio at the bottom of MgO and CoFeB /MgO interface before radiation is just right, which is close to the perfect MgO crystal.

After radiation with 10 Mrad(Si), the peak of Mg 1 s at 15 s is 1304.2 eV, and the peak of Mg 1 s at 20 and 25 s is reduced by 0.2 and 0.7 eV, respectively, to 1304.0 eV and 1309.5 eV. Therefore, the distribution of O remains unchanged after radiation, that is, there are fewer O atoms at the bottom and CoFeB/MgO interface, while there are more O atoms at the top and middle of MgO. However, the Mg 1 s peaks at 15, 20, and 25 s after radiation decrease by 0, 0.2 and 0.4 eV, respectively, compared with that before radiation. From thetop to the middle and then to the bottom, the peak position decreases more and more, indicating that the O atoms have diffused from the interior (middle and bottom) of MgO to the CoFeB /MgO interface, and the hypoxia at the bottom of MgO is more serious, and oxygen vacancies are created at the bottom of MgO.

**Figure 8:**
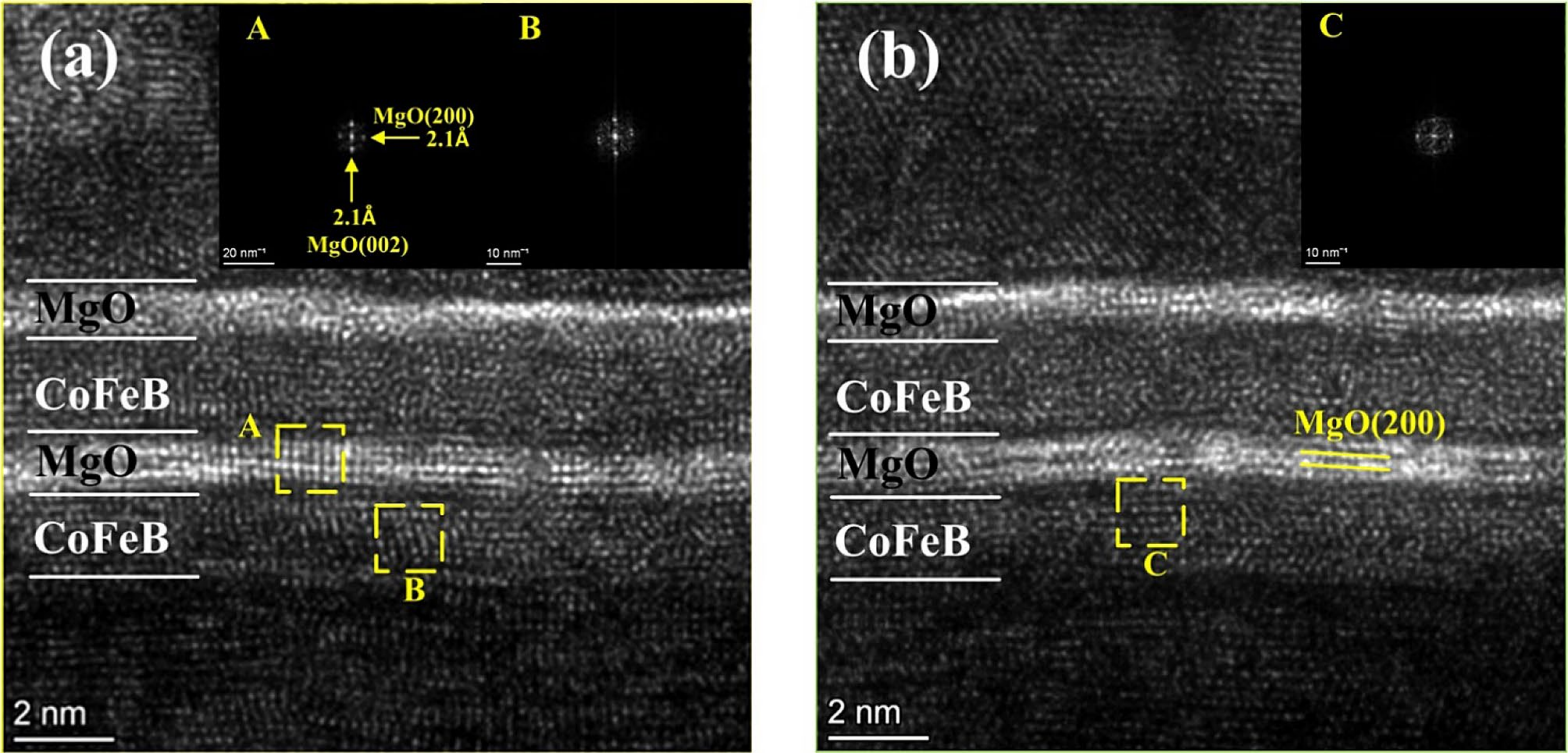
HRTEM image of MTJ devices (**a**) without radiation and (**b**) with 10 Mrad(Si) radiation.

We use HRTEM to study the effect of high dose X-ray radiation on CoFeB/MgO/CoFeB crystal structure, especially MgO crystal structure. As shown in Fig. 8a, before radiation, the MgO layer crystallizes and has a very uniform (200) orientation. This indicates that the crystal structure and quality of MgO are good before radiation. After 10 Mrad(Si) radiation, the MgO tunnel barrier layer becomes partially amorphous. Combining HRTEM and XPS results, we may conclude that oxygen atoms are stimulated due to X-ray radiation and thus oxygen vacancies are formed in the MgO layer. Few oxygen vacancies have little influence on the switching property of magnetic tunnel junctions. As the density of oxygen vacancy increases, the conducting path may be formed due to the redistribution of oxygen vacancies when we sweep the R-V curves. Once the radiation dose exceeds a certain value, the conducting path may be formed, and thus the MgO breaks down during radiation. So all experimental MTJs have only a resistance value of about one hundred ohms after 10 Mrad(Si) radiation.

In summary, the total ionizing dose effect of X-ray radiation on the current induced magnetization switching performance of CoFeB/MgO/CoFeB magnetic tunnel junctions has been investigated. It shows that the critical switching current and TMR of MTJs change slightly after 8 Mrad(Si) radiation. However, the deterioration and breakdown characteristics of the CIMS property are observed, which is attributed to the degradation and breakdown of MgO. The XPS and HRTEM results indicate that the X-ray radiation gives rise to oxygen vacancies that may redistribute in the CIMS sweep and thus form a conductive path in MgO.

## Methods

### Sample preparation

Ta (10)/Ru (2.5)/Pt (2.5)/[Co/Pt]_5_/W (0.25)/CoFeB (1.8)/MgO (1) /CoFeB (2)/MgO (1)/Ru (2.5)/Ta (10) (unit: nm) were deposited on Si/SiO_2_ substrates by magnetron sputtering in a base pressure of 3 × 10^−7^ Torr at room temperature. The Ta, Ru, Pt, Co, W, and CoFeB layers were deposited by DC sputtering and the MgO layer was deposited by RF sputtering under an Argon pressure of 3mTorr. After the deposition of the multilayer stack, the MTJ structures were patterned elliptical shape with a junction area of 60 × 150 nm^2^ (minor × major axes) using photolithography and ion milling. The MTJs were thermally annealed at 350 °C for 30 min in a vacuum furnace with a base pressure of 3 × 10^−7^ Torr. In order to distinguish the chemical state changes at CoFeB/MgO and MgO/CoFeB interfaces, we also deposited Ta(10)/CoFeB(1.8)/MgO(1)/Ta(3) (unit: nm) and Ta(10)/MgO(1)/CoFeB(2)/Ta(3) (unit: nm), respectively.

### X-ray radiation experiment

The X-ray radiation experiments were performed using an X-ray radiation source system (GX200). The X-ray is emitted from an X-ray tube and burst through an optical gate onto the MTJ die. During the X-ray radiation, the X-ray tube voltage and current were set to 40 kV and 25 mA. The CIMS curve of MTJ devices in the experimental group was characterized by Semiconductor Device Analyzer B1500 before and after radiation exposure. The X-ray emitted by the X-ray tube forms a uniform radiation spot after collimation. The dose rate was a constant 494.7 rad(Si)/s. The experimental samples finally received a cumulative 10 Mrad(Si) by three dose stages of 8 Mrad(Si), 9 Mrad(Si), and 10 Mrad(Si). After each dose stage is completed, the X-ray radiation equipment is shut down and CMIS curves were performed.

### Microstructural investigation

The interfacial chemical states were investigated via X-ray photoelectron spectrometry (XPS, PHI Quantera II). The Al Kα line at 1486 eV was used. The multilayer samples were sputtered using a 2 keV Ar^+^-ion gun to remove the cap layer and the other layers. The Ar gas pressure was 2 × 10^−5^ Pa and the diameter of the Ar^+^-ion beam was 200 um. XPS data were detected at a 90°take-off angle for photoelectrons with respect to the sample surface. Then, Ar^+^-ion etching XPS was used to study the multilayer films at various depths. All binding energies were calibrated to eliminate the charging effect with C 1* s* (284.6 eV) and Ta 4*f*_7/2_ (21.7 eV). The microstructures of MTJ nanopillars were characterized by a high-resolution transmission electron microscopy system (HRTEM, Talos F200X).

## Data Availability

The datasets generated during and analyzed during the current study are available from the corresponding author on reasonable request.
